# Using Voice and Touchscreen Controlled Smart Speakers to Protect Vulnerable Clients in Long-Term Care Facilities

**DOI:** 10.1093/geroni/igac024

**Published:** 2022-04-15

**Authors:** Joan K Davitt, Jocelyn Brown

**Affiliations:** School of Social Work, University of Maryland, Baltimore, Maryland, USA; School of Social Work, University of Maryland, Baltimore, Maryland, USA; School of Medicine, University of Maryland, Baltimore, Maryland, USA

**Keywords:** COVID-19 pandemic, Guardianship, Residential care, Technology, Vulnerable adult

## Abstract

**Background and Objectives:**

The Centers for Medicare and Medicaid Services restricted long-term care facility visitation to only essential personnel during the coronavirus disease 2019 pandemic. The Maryland Department of Human Services distributed Amazon Echoshow 8 voice and touchscreen controlled smart speakers (VTCSS) to a sample of their institutionalized guardianship clients to enhance caseworker access during the pandemic.

**Research Design and Methods:**

This pilot study focused on understanding VTCSS use challenges and the effects on clients’ safety and well-being. Two focus groups were conducted with caseworkers (*N* = 16) who piloted the devices. The interviews were recorded, transcribed, and analyzed using open and axial coding.

**Results:**

Four themes were identified, including challenges to providing casework during the pandemic (e.g., facility technology gaps), challenges to device installation and use (e.g., privacy concerns), strategies for overcoming challenges (e.g., alert features), and benefits (e.g., stimulation, care monitoring) and uses (e.g., enhanced access, entertainment).

**Discussion and Implications:**

VTCSS show great promise to engage the client, maintain visual access, and monitor quality of care. However, facilitating access to such technology requires planning and training before installation.

**Translational Significance:** This pilot provided voice and touchscreen controlled smart speakers (VTCSS) to guardianship clients in long-term care (LTC) facilities during the pandemic to enhance caseworker access. Findings suggest these devices are assisting caseworkers in connecting with their clients and in providing client stimulation when direct access is limited. Challenges included: low facility staff technology literacy, lack of Wi-Fi access in some facilities, and concerns related to resident privacy. This study shows VTCSS have potential to benefit vulnerable LTC residents and their families enhancing access and engagement, facility staff by reducing staff burden, and access to support services to promote resident well-being.

## Background and Objectives

The coronavirus disease 2019 (COVID-19) pandemic has had a profound impact on individuals living and working in long-term care (LTC) facilities (Chen et al., 2020; Thompson et al., 2020). Older adults and people with intellectual and developmental disabilities (IDD) are particularly physically, socially, and mentally vulnerable to the effects of the pandemic. Residents of LTC facilities are more likely to experience heart and respiratory conditions, making them more susceptible to the virus and increasing the risk of death (Courtenay & Perera, 2020; Thompson et al., 2020). LTC residents account for 40%–50% of COVID-19 deaths but make up only 6% of cases (Lau-Ng et al., 2020; White et al., 2021).

Due to high transmission and death rates in LTC facilities, in March of 2020, the CMS directed facilities to restrict nonessential visitors, cancel group activities, and require residents to eat in their rooms (CMS 2020a). Between March and September of 2020, LTC facility residents were prevented from congregating or socializing with their family, friends, or other residents (Edelman et al., 2020; McArthur et al., 2021; Mo & Shi, 2020). In September of 2020, some restrictions were eased but not eliminated. LTC facility residents experienced disruptions in daily routines, activities outside of the facility, and social interactions with family and friends (Conroy et al., 2020; Sheerman et al., 2020), worsening social isolation in older adults (MacLeod et al., 2021).

To enable caseworker access to their guardianship clients and enhance the quality of care monitoring, the Maryland Department of Human Services (DHS), piloted the Echoshow 8 voice and touchscreen controlled smart speakers (VTCSS). These devices have HD smart display with Alexa (voice commands) and 13 MP camera and require only Wi-Fi connection and power, making them relatively easy and inexpensive to use. Clients with visual impairments can use the device through voice commands, while those with hearing impairments can use the captioning and touchscreen control. The larger screen, lower purchase cost, and lack of monthly access fees make these better alternatives than smartphones. DHS had two key goals for this initiative. First, to enable caseworkers to visually connect with clients during the pandemic lockdown to better monitor client well-being. Second, to help clients connect to family/friends, and to the outside world, via the internet to reduce social isolation and maintain engagement. The pilot study focused on understanding the device benefits for clients, caseworkers, and facilities and implementation challenges.

Wu (2020) and Simard and Volicer (2020) define social isolation as an objective state with few relationships and infrequent social contact. Social isolation can be conceptualized as a deprivation of social connectedness (Zavaleta et al., 2017) or the extent to which an individual cares about other people, feels cared about by other people, and feels as though they belong to a group or community (O’Rourke & Sidani, 2017). Low social connectedness can result in social isolation, which is a risk factor for loneliness (O’Rourke & Sidani, 2017; Simard & Volicer, 2020; Wu, 2020). Isolation, loneliness, and lack of social connectedness are negatively associated with physical and mental health, increasing the risk of hypertension, weight gain, depression, anxiety, heart disease, cognitive impairment, Alzheimer’s disease, and death (Bethell et al., 2021; Cacioppo et al., 2016; Cornwell & Waite, 2009; Dinapoli et al., 2014; Holt-Lunstad et al., 2015; Sheerman et al., 2020; Valtorta et al., 2016; Wu, 2020).

The prevalence rate of social isolation and loneliness for LTC facility residents is at least double compared to community dwellers; however, social isolation and loneliness have been exacerbated during the pandemic (Elias, 2018; Simard & Volicer, 2020). Distancing protocols have interrupted the frequency and quality of resident social connectivity creating a “connectivity paradox” where the most vulnerable are forced into isolation to protect them from the virus (Smith et al., 2020, p. 2).

LTC facility residents are also at increased risk of boredom due to lack of stimulation (Bolt et al., 2021; Brown et al., 2020; Kolanowski et al., 2019). Liddell et al. (2021) found that boredom is associated with sedentary or restless behavior, agitation, apathy, and lack of stimulation with increased risk of wandering, particularly in residents with dementia or intellectual disabilities. Such challenges are exacerbated during facility lockdowns because residents are unable to understand the purpose of distancing measures (Liddell et al., 2021).

Information communication technologies (ICTs) are a broad category of digital communication devices and applications including email, the internet, VTCSS, smartphones, and virtual meeting platforms. Prior to COVID-19, ICT use was primarily initiated by LTC facility residents to communicate with friends, family, and supports. Residents have also used ICTs for entertainment (e.g., watching movies), stimulation (e.g., puzzles), and information (e.g., news; Baker et al., 2018; Blaschke et al., 2009; Boeder et al., 2020; Chen & Schulz, 2016; Demiris et al., 2008). Several studies found that ICTs reduce social isolation and loneliness and increase stimulation and social connectedness in LTC facilities (Baker et al., 2018; Chen & Schulz, 2016; Edelman et al., 2020; Ibarra et al., 2020; Kluck et al., 2021). Social media services (e.g., Facebook), email, video chat (e.g., FaceTime), and messaging services (e.g., WhatsApp) were the most common interventions evaluated in LTC facilities (Baker et al., 2018; Ibarra et al., 2020). However, methodological and measurement differences across studies require further research to fully understand the benefits of ICTs (Baker et al., 2018; Chen & Schulz, 2016).

ICT prevalence, uses, and types vary significantly across LTC facilities in the United States (Powell et al., 2019). Research shows that, generally, LTC facilities do not possess the necessary ICT infrastructure due to the lack of staff with technological skills, funding, and Wi-Fi capability (Gallistl et al., 2021; Moyle et al., 2018). Powell et al. (2019) found that 86% of nursing homes provided residents with no access to technology.

For adults aged 60 and older, the use of technology continues to grow significantly (Kakulla, 2020). The most common form of ICT used by older adults are smartphones (62%–81%) and tablets (40%–49%), which they use for email (67%–70%), internet browsing (56%–63%), weather (69%–70%), photos (55%–64%), and social media (50%–57%). Although early research suggests that individuals with IDD are less likely to use ICTs, like computers, tablets, and smartphones, little research has been done on newer technologies like VTCSS (Carey et al., 2005; Carmeli et al., 2004; Tanis et al., 2014).

VTCSS have the potential to transcend physical and cognitive barriers unique to the aging and IDD populations (Kim, 2021; Masina et al., 2020; Pradhan et al., 2018). For example, older people with arthritis may find the use of smartphones and tablets cumbersome and possibly painful while someone with visual impairments may not be able to engage with these devices at all. O’Brien et al. (2020) established that older adults primarily use VTCSS for entertainment, companionship, and reminders while Jones (2019) suggests that VTCSS allow activities that people were previously unable to do. Elza et al. (2017) found that VTCSS improves social interaction, subjective social support, and loneliness. Also, VTCSS were found to be, suitable for those with a range of abilities and particularly useful to those with visual impairments (Pradhan et al., 2018).

LTC facilities were under immense pressure as the pandemic raged (Kirkham & Lesser, 2020). White et al. (2021) found direct care staff were exhibiting burnout due to increased workloads, staffing shortages, and providing care for socially isolated residents. However, facilities are responsible for ensuring that residents are safe and receive quality care despite staff turnover or burnout (Berg-Weger & Morley, 2020). As such, it is timely to explore how VTCSS could be used to ensure quality care and resident well-being during and beyond the pandemic. This study reports the results of the pilot phase of a comprehensive evaluation of the Echoshow 8 Initiative in selected facilities in one state. This initiative, directed at some of the most vulnerable and socially isolated LTC residents, has broad implications for improving quality of life and connectedness.

## Design and Methods

This pilot focused on understanding the potential challenges and benefits of using these devices and strategies for assuring smooth implementation of the devices in the statewide rollout. Caseworkers were purposely recruited by the Adult Public Guardianship (APG) Program Specialist to assure adequate interest in using technology to facilitate client access and a willingness to act as county “champions” during the statewide rollout. The APG Program Specialist recruited participants during their monthly trainings and through email. Sixteen caseworkers volunteered to pilot the devices with their guardianship clients. The caseworkers, representing 14 of 23 counties, were trained on: account set-up on agency-issued cell phones, device set-up, and device use with their clients and LTC staff. All caseworkers were women, with MSW degrees that had worked in adult services for at least 2 years.

Sixteen case managers participated in two virtual focus groups, one before and one after installation of an Echoshow device, installed with at least one of their clients. All clients lived in some form of LTC facility including nursing homes, assisted living facilities, and small group homes. The sample included caseworkers responsible for clients with a variety of diagnoses and physical, behavioral, and cognitive challenges that we note in the results. The interview guide included questions on challenges to doing casework during the pandemic, challenges to installing and operating the devices, benefits of the Echoshow 8, and strategies for troubleshooting challenges. The focus groups were conducted virtually by the study Principal Investigator immediately after device training meetings for caseworker convenience. Both the PI and research assistant attended both meetings and took notes. The group interview process inspired participants to freely share their experiences; coparticipants frequently concurred with their peers’ examples.

Interviews were recorded by Google Meet and transcribed verbatim using Otter.ai software. Transcriptions were reviewed for accuracy by the PI which began the data immersion and familiarization process. Using open coding and not guided by any framework, the transcript of the first interview was reviewed by the PI to develop a coding scheme and to organize the open codes into broader categories (axial coding; Nelson & Poulin, 1997). Each researcher independently reviewed both transcripts using the initial code-set and new themes were added as needed. The categories were consistently validated against the data to test emergent themes and alternative explanations (Marshall & Rossman, 1999). To enhance analytic trustworthiness, any disagreements were resolved via a joint review and consensus process (Lincoln & Guba, 1985).

## Results

Most participants agreed with many of the issues/concerns discussed by their peers, with folks nodding, sharing their agreement in the chat feature, or stating their agreement. Major themes are discussed later with participant quotes to highlight the theme.

### Challenges to Providing Casework During the Pandemic

The initial question asked the caseworkers to describe the challenges they were having providing casework services during a pandemic where LTC facilities were operating on complete lockdown. In many instances, caseworkers had not seen their clients for the entirety of the lockdown, or they were using cell phones (e.g., facetime) to attempt visual access. All caseworkers discussed how difficult it was to physically see their clients during this time, even if their client was able to use a cell phone or email.

I have not done visits since the pandemic. I normally would go out monthly or twice a month. It’s been a real challenge for me wondering how they’re doing. I get emails and emails are ok but I want to, lay eyes (on them).

Most caseworkers noted that many of the facilities had challenges using technology to help with video access to their clients. Either they do not use cell phones, do not know how to use them, or have limited Wi-Fi capability such that ICTs are not supported.

Some of the more seasoned assisted living providers are not up to the technology. The nursing homes some of the younger staff (can do) a video call on their phone. Some of the older care providers, they don’t have Wi-Fi; they don’t know how to really work their personal phone.

They also expressed concerns about having to wait for the right staff person to be on duty to use technology. Moreover, there are Maryland DHS restrictions on the types of software caseworkers are allowed to use. One caseworker expressed their collective frustration when facility technology did not align with agency technology. “I would have to wait until a certain person was on shift for them to work with the technology that DHS allows. A mismatch of technology, that would prevent me from making visits.” Not being able to see their clients was a major concern for all of the caseworkers. They did not trust relying on facility staff for client updates. This was particularly problematic if they had clients that had no or limited verbal abilities.

I have one client in a persistent vegetative state and his parents are here locally although they live in Florida. They haven’t been able to visit since COVID. It’s frustrating; I have asked the nursing home for video communication for myself and the family. It’s really important that they see him.

Several caseworkers noted that they could no longer visit some clients at their day programs because these had also shut down. This was an important way that caseworkers could see their clients away from the watchful eye of LTC facility staff. This also meant that residential care staff now had to keep their residents engaged and provide stimulating activities throughout the day. One caseworker captured the sentiment of all in this way: “the lack of structure in the home is causing additional stresses for some providers. I have clients who are regressing. By the time COVID is over, we will have to start from the beginning.” Several others noted that their clients did not respond well to phone contacts, and in many cases this was the only means to engage with their client.

on the phone she was very short, wasn’t talking very much. She wasn’t recognizing my voice or able to see me at all. It wasn’t a good contact. Her residential staff said that she has been fine but I was concerned (because), she’s usually not that short or agitated.

Another caseworker similarly noted:

They have fear, paranoia, not really knowing who they’re speaking with. When I would visit her face-to-face she told me everything. But over the phone, she’s hesitant. “Is this really you? Who am I talking to?”

Finally, in cases where the facility staff could not master the technology, some caseworkers had to resort to in-person visits outside of the facility wearing PPE to keep everyone safe. “I’m seeing those clients by going to the porch, gowning up, and having them put on everything. The challenge is the risk of still doing home visits because the care provider is not technology savvy.”

### Challenges to Device Installation and Ongoing Use

One of the biggest challenges to device installation and use was the need for facility staff help since the caseworkers were not allowed in the facility. Many facilities did not have staff that understood technology, and this made it difficult to get the devices installed in some facilities. “My biggest concern is the majority of my providers are not tech-savvy. They’re not even able to operate their cellphones.” Even for something as simple as entering a password to set up the device, some caseworkers had to assist the facility staff. One caseworker noted: “They did an extension cord out, brought the device outside so I could input the password.” Additionally, caseworkers had to depend on staff to demonstrate device operation to the clients; when facility staff was overwhelmed, there were delays in setting-up devices. “The nursing home has not even set it up yet. It’s been a week. That’s infuriating because I keep checking, and it’s still offline.”

Caseworkers sometimes needed staff to troubleshoot problems once the device was installed, such as dropped Wi-Fi connection or showing the client how to answer the device. For example, in one nursing home, “the issue was that it would constantly disconnect from the Wi-Fi and to get them to reconnect took days. They would say ‘oh yeah, I’ll get to that’.” Moreover, some smaller facilities did not have Wi-Fi available. “I have some very small assisted livings and they don’t have Wi-Fi. Affordability, they have no reason to have WI-FI.”

Several caseworkers described device use challenges related to other residents within the facility. Before installation, some facilities expressed concerns about installing such devices in a client’s room and how that might affect the privacy of a roommate or other residents. “I just called the nursing home and they said ‘no that’s privacy because she’s in the room with someone else’.” Caseworkers also worried about how to keep other residents from using the clients’ Echo device. Since this is a voice-controlled device, activated by saying “Alexa,” other residents could potentially use the device without the client’s permission or knowledge. “It’s going to be hard to not allow everyone who’s in the home to use it. I just don’t see us being able to regulate that as long as our client has access to it when they need it.” Some facilities adamantly refused to allow device installation.

During the pandemic, no one cared for her hair. Her hair is now mangled and they wanted to shave her hair off because of their lack of care. There’s places (like this) that I would love for these devices to be so I would know that my clients are being cared for.

Security of the device was a concern for many caseworkers. “Mine would definitely be making sure (the device is) secure, that it’ll be there, overnight, they have different staff coming in.”

Caseworkers also expressed concerns related to the clients and their use of the device. Caseworkers had to work with clients to help them understand how to use the device. This was challenging because they could not enter the facilities and had to guide the client via a cell phone, in some cases without staff support. In addition, there were concerns about clients using the device to access inappropriate content. One caseworker asked, “can we set boundaries for some of our clients (with) inappropriate behaviors; I’m concerned that they would access sites that we wouldn’t want?” Another concern was if some clients might be stressed or upset by the devices. “Possible paranoia with the cameras. They’ll probably feel like we’re tracking them with these devices, following their movements.” Another caseworker noted: “I’m thinking of non-verbal clients. Let’s say the staff member walks away and they become unsettled with what’s going on with the device and not being able to turn it off and becoming distressed.” Caseworkers also raised concerns about the possibility of a client breaking their device.

The caseworkers discussed using the devices to drop-in on their clients unannounced to check on the care the client was receiving. One caseworker noted that facility staff raised concerns about resident privacy. “He (facility staff) said maybe you could call ahead of time and let us know you want to reach her. But that defeats the purpose because it’s nice for us just to drop in (unannounced).” All caseworkers discussed if unannounced virtual visits could be an invasion of the client’s privacy. One caseworker pointed out:

When I go to the client’s facilities, the staff usually either take me to them or make sure they’re not busy. Being able to see them in 10 seconds, they might not even have a chance to put on clothes. I can see a concern with that.

Another worker said: “It is a bit invasive. If you went to somebody’s home, you’re gonna knock on their door, you’re gonna knock on the resident’s bedroom door, rather than just barging in.”

Other challenges included caseworker ability to operate the device, creating a separate amazon account, and navigating device features. For example, one caseworker had trouble with a facility changing the settings. “When I went to call her it said Do Not Disturb when I pulled it up on my phone.” Another caseworker raised concerns about having more than one client with a device and how the system would handle alerts or announcements. “One of the biggest issues is when you have more than one device, and you’re doing drop-ins (messages), it’s going to all devices.” In addition, since amazon tracks usage, the caseworkers worried about having two devices with two very different clients. “If I have a 22-year-old girl and a 60-year-old-man, they’re going to get suggestions for each other because that account is connected, even though they have two separate devices.”

Moreover, all caseworkers wondered if they could use the call feature of the device to connect clients with other social services, such as the client’s nurse or attorney. The devices, however, can only provide audio connections when being used to call someone without an Echo/amazon device. Also, when the client calls someone using the Echo, the caseworker’s number appears. “In theory, the person with the device could be calling anybody, but it would look like it (was) coming from us (the caseworkers).”

### Overcoming Challenges

Various strategies were identified to address some of the challenges. The state was working on obtaining headphones so that clients with a hearing impairment could still use these devices, and it might reduce privacy concerns as well when others in the facility would not be able to hear both sides of the conversation. Furthermore, the caseworkers realized they could use the announcement feature to let the client know they would be calling or dropping in shortly. One caseworker noted: “There is a way you can have a message pop-up on their device that says Kim is gonna call you in 10 minutes or drop in at 10:30. They would know to prepare.” Other options to address privacy challenges included the client can turn the camera off, accept the call without video, or simply decline the call. “The drop-in (feature) blurs the screen for the first 10 seconds, so the person can decline it.” Some caseworkers were not comfortable with using the drop-in feature, for example, “I think I’m still of the mindset that I will call first to let them know because that’s her private room.” Another caseworker suggested that they talk to their respective clients regarding the drop-in feature and how to handle this, which would give some control back to the client but still enable the caseworker to see the client without the facility being alerted to the visit. These strategies (summarized in Table 1) would protect privacy while caseworkers get an accurate idea of the care the client is receiving.

**Table 1.** Comparison of Benefits, Challenges and Strategies of Voice and Touchscreen Controlled Smart Speakers (VTCSS)

Benefits	Challenges	Strategies
Functional for residents with differing abilities	Concerns about whether devices might generate negative reactions for some residents (e.g., those with paranoid tendencies)	Caseworkers must work closely with facility staff to identify the best clients/ residents to have such a device
Provides entertainment for residents (e.g., watching movies or you tube video) Provides stimulation for residents (searching for information, weather, news)	Concerns about residents accessing inappropriate content via the device	Parental controls can be set to prevent accessing inappropriate content
Enhances engagement (with outside world via the internet; with family/caseworker)	Inability to limit device access to just the target resident	Need to work closely with facility staff in terms of where to locate the device to limit access Also, device may be used to benefit several residents in relation to stimulating and engaging activities
Enables caseworker to monitor client status and quality of care	Resident privacy (both the target resident and other facility residents)	Use of device drop-in and announcement features can reduce privacy concerns. Discussing with client in advance as to how they wish to handle drop-in features
Could enhance access to other support services.	Device loss or damage	State issued letter explaining the vetting process and device review and approval. Also, releasing facility from liability if device is lost or damaged
Protect residents and caseworkers from communicable illness such as COVID-19	Lack of Wi-Fi access in some facilities raises equity concerns	Will need to develop policies and resources to support particularly smaller facilities to provide Wi-Fi access to their residents.
Reduce facility staff burden during facility lockdown by keeping residents engaged	Need for facility staff intervention with device during lockdown. Facility staff lacking technology savvy	Need to provide facility staff with training on the device features, operation, and navigation
Enhance caseworker productivity and efficiency	Many caseworkers may not be technology savvy	Caseworkers need access to work smartphone to make this effective and training on setting up separate amazon account, device operation and navigation

## Benefits and Uses

Caseworkers were creative in finding ways to make it work with a variety of clients with differing abilities, including clients with hearing, visual and speech impairments, intellectual, developmental, or cognitive disabilities.

I have a client who is considered legally blind and he would really enjoy interaction with the device, asking for information or a joke because he’s isolated to his room. The voice interaction part will be very helpful for him because having to push buttons or dial a number is very difficult but (voice activation) will really open up opportunities to gain stimulation and satisfaction.

One caseworker noted changes that she made to enable a client with a speech impairment to still use the device. “He can’t say Alexa in a way that (the device) can understand. We switched (the name) to echo and he can say that just fine.” Another worker noted: “one of the reasons I thought this would be really good is because she’s diabetic, also prone to wounds. I can sometimes see how she’s doing just by the color of her skin.” Even clients that are nonverbal could use the device because it is also touchscreen activated. For example, “about half of my clients are non-verbal. I would be comfortable just being able to see them, and they can hear my voice and respond with a facial expression or eye contact.”

Caseworkers also identified multiple ways the clients could benefit from using the devices, such as checking news or weather or general entertainment. One caseworker noted: “since our clients can’t go to the day programs they’re extremely bored … these devices will give them another venue to ‘research’, read, watch videos, to decrease their boredom and keep them connected.” Another noted regarding one of her clients: “One gentleman in the nursing home is very bored because he doesn’t have a computer anymore; this will give him the opportunity to learn things. I’m excited for him.” Another discussed her client: “she does not sleep at night. And I would rather her listen to music (via the device), than unfold and refold her clothes, which is what she’s been doing to keep herself occupied.”

The devices might also provide an opportunity for other health/support service providers to see the residents. One caseworker commented: “We have clients that have non-life-threatening health issues and they’re not able to get treatment because facilities won’t send anybody out. It might be helpful to get video visits to places that normally wouldn’t have them.” Another worker noted: “I would love for his lawyer to see what I saw. This client came from a really bad situation. We both were really nervous all these months.”

Clients can use the device to stay in touch with family/friends. One worker noted:

She’s got a sister who lives in another state she just has a flip phone. To get them to communicate in a private way has been really hard. I set her sister’s name up in my phone. And I told her to call her when we got off. I was excited about the device because it would be great for family members to have this (for) visits.

The devices can also help to protect the clients, caseworkers, and the entire facility community because caseworkers can visit remotely during the pandemic. One worker noted: “I’m excited about that (device) so I can stop going out and risking everybody in the house.”

All caseworkers discussed how these devices could help the facility staff with engagement or support. One caseworker noted: “I have individuals that, I’m their only contact. When they need a correction for their behavior, the staff call me. This might be easier to shift, if they can see instead of just hear me.”

All caseworkers noted how such devices could improve their efficiency and productivity. “This technology would be helpful to have longer visits.” Another noted: “That’s going to help a whole lot in terms of travel. I had clients far away in the southern part of the state and this would have helped.” Another said: “This would be an excellent tool to use in inclement weather.” Simply being able to see their clients in times when they are unable to get into the facilities, was a tremendous relief for all caseworkers. “I could see inside the assisted living, you saw it was clean, he was dressed. I saw the staff had on all their PPE.”

## Discussion and Implications

This study suggests that access to guardianship clients in LTC was a critical problem during the pandemic. Many facilities lacked the technology, fiscal resources, or technological literacy to offer innovative solutions to ensure their vulnerable residents remain engaged. The caseworkers discussed examples of client deterioration or poor care during the pandemic lockdown. Residents in certain types of facilities may be at greater risk of deterioration during such crises due to a lack of resources and knowledge. This was especially true for smaller group homes, but even large nursing homes struggled to provide access during the pandemic lockdown. Which facilities have access to technology to enable visual and audio access, is a question of equity. Resistance to installing Wi-Fi and/or devices may negatively affect some residents access to the outside world during lockdowns.

This pilot study is consistent with previous research that ICT can work for a variety of clients (Ho, 2018; Jones, 2019; Kim, 2021; Masina et al., 2020; O’Brien et al., 2020; Pradhan et al., 2018), while also benefitting caseworkers, facilities, and clients’ families. Although, we were unable to measure these constructs directly, these results align with previous research on technology’s ability to reduce isolation or loneliness and provide stimulation, engagement, and entertainment for older adults and those with IDDs (Boeder et al., 2020; Demiris et al., 2008). Although we were unable to interview facility staff, such devices have the potential to enhance resident cognitive engagement. However, some involvement of facility staff will be required. Similar to previous findings (Cavallo, 2015), this study shows that technology can be used to ensure clients were receiving quality care as devices provided caseworkers visual access to their clients. Unlike the current literature, however, this study established that VTCSS assisted caseworkers in working remotely during a global pandemic. Moreover, VTCSS have the potential to enhance the efficiency of casework and may reduce the risk of illness when either party is sick. Moreover, VTCSS may be used to increase families’ access to their loved ones, which can also improve care quality and overall resident well-being.

There are several advantages to using VTCSS over other devices such as smartphones and tablets. VTCSS devices are voice-activated, ideal for individuals with visual or reading impairments. They are also touchscreen controlled and have captioning options, beneficial to those with hearing impairments. VTCSS can also assist those with physical limitations in using digital interfaces that would otherwise require manual navigation. For caseworkers, these devices provided the ability to drop-in on clients with physical and cognitive limitations or were uncomfortable using other forms of technology such as cellphones. For example, several caseworkers remarked that clients were resistant to talking by phone. The stationery and voice-controlled nature of the device can circumvent limitations while providing visual access to clients, often, independent of staff assistance.

Two key ethical issues raised by caseworkers focused on the well-being and privacy of facility residents. The use of such devices to check on residents can be important in terms of monitoring the quality of care a resident is receiving. However, there is a risk of compromising resident privacy. Caseworkers were able to develop procedures, such as using announcements or drop-in alerts or calling ahead by phone to address privacy concerns for their clients. However, there is still an issue of how to protect the privacy of other residents in the facility. The possibility for these devices to have a negative impact on the clients’ or other residents’ mental or physical health is another concern. For example, the devices could exacerbate paranoid ideations for residents leading to distress. Caseworkers will need to work with facility staff to determine which clients would benefit the most from these devices, the safest and least invasive location for the devices, and strategies for use that will not upset nor compromise the clients or other residents.

A major challenge to device implementation in this evaluation was the facilities’ lack of technological knowledge or access to the internet. If research continues to demonstrate benefits from VTCSS for enhancing access to and quality care for LTC residents, funding may be necessary to enable smaller, under-resourced facilities to provide Wi-Fi for resident use. Some staff and caseworkers may be more technology literate than others. Thus, all involved will need training on device set-up and use, including caseworkers, facility staff and volunteers, and residents. Training should also include instructions on what types of residents/clients may benefit or not from such a device. This type of approach works best when the caseworkers have state or agency-issued cell phones and can set up accounts separate from their private accounts.

The department overseeing implementation will need to develop assurances for facility management related to device use, damaged or lost devices, client privacy, etc. to address facility concerns and counterresistance. In this project, the Attorney General’s office issued a letter assuring facilities that the state had thoroughly reviewed the technology, approved the devices for use, and facilities would not be liable for lost or damaged devices. It would be wise to include facilities in the planning process for both their expertise and to address resistance.

### Limitations

As with all research, this pilot efficacy evaluation has some limitations. First, we were unable to measure resident outcomes and thus cannot determine device effects on resident quality of life or well-being. The study was conducted in one state, with guardianship clients only and a small sample of volunteer caseworkers. Such a sample without a comparison group is subject to response bias. We were unable to triangulate with data from multiple sources to evaluate the validity of the results (Carter et al., 2014). However, the goals of this initial phase were to determine if device use was feasible within a LTC setting, what types of guardianship clients might benefit from device use and how, and best practices to implement statewide device distribution within the guardianship program. A formal evaluation to assess outcomes is currently underway as devices are being distributed to guardianship and other adult services clients throughout the state. Future research should continue to evaluate specific benefits to device use for vulnerable adults in LTC, utilizing increased sample sizes, control or comparison groups, and data triangulation or replication to control for threats to internal validity.

## Conclusion

VTCSS show great promise in their ability to engage the resident, provide visual access, monitor quality of care, and enhance connectedness. VTCSS has tremendous potential to benefit vulnerable LTC residents when implemented in a structured program.

## References

[CIT0001] Baker, S., Warburton, J., Waycott, J., Batchelor, F., Hoang, T., Dow, B., Ozanne, E., & Vetere, F. (2018). Combatting social isolation and increasing social participation of older adults through the use of technology: A systematic review of existing evidence. Australasian Journal on Ageing, 37(3), 184–193. doi:10.1111/ajag.12572 30022583

[CIT0002] Berg-Weger, M., & Morley, J. E. (2020). Loneliness and social isolation in older adults during the COVID-19 pandemic: Implications for gerontological social work. Journal of Nutrition, Health and Aging, 24(5), 456–458. doi:10.1007/s12603-020-1366-8 PMC7156792 32346678

[CIT0003] Bethell, J., Aelick, K., Babineau, J., Ba, M. B., Edwards, C., Ba, J. G., Hewitt, D., Rn, C., Iaboni, A., Lender, D., Schon, D., & Rn, K. S. M. (2021). Social connection in long-term care homes: A scoping review of published research on the mental health impacts and potential strategies during COVID-19. Journal of the American Medical Directors Association, 22(2), 228–237.e25. doi:10.1016/j.jamda.2020.11.025 33347846 PMC9186333

[CIT0004] Blaschke, C. M., Freddolino, P. P., & Mullen, E. E. (2009). Ageing and technology: A review of the research literature. British Journal of Social Work, 39(4), 641–656. doi:10.1093/bjsw/bcp025

[CIT0005] Boeder, J., Hwang, S., & Chan, T. (2020). Engagement with life among the oldest-old in assisted living facilities: Enriching activities and developmental adaptation to physical loss. Ageing and Society, 42(5), 1–22. doi:10.1017/S0144686X20001488

[CIT0006] Bolt, S., van der Steen, J., Mujezinovic, I., Janssen, D., Schols, J., Zwakhalen, S., Chandni, K., Knapen, E., Dijkstra, L., & Meijers, J. (2021). Practical nursing recommendations for palliative care for people with dementia living in long-term care facilities during the COVID-19 pandemic: A rapid scoping review. International Journal of Nursing Studies, 113, 1–13. doi:10.1016/j.ijnurstu.2020.103781 PMC7526658 33080475

[CIT0007] Brown, E., Kumar, S., Rajji, T., Pollock, B., & Mulsant, B. (2020). Anticipating and mitigating the impact of the COVID-19 pandemic on Alzheimer’s disease and related dementias. American Journal of Geriatric Psychiatry, 28(7), 712–721. doi:10.1016/j.jagp.2020.04.010 PMC7165101 32331845

[CIT0008] Cacioppo, S., Grippo, A. J., London, S., Goossens, L., John, T., & Development, A. (2016). Loneliness: Clinical import and interventions. Perspectives on Psychological Science, 10(2), 238–249. doi:10.1177/1745691615570616.Loneliness PMC4391342 25866548

[CIT0009] Carey, A. C., Friedman, M. G., & Bryen, D. N. (2005). Use of electronic technologies by people with intellectual disabilities. Mental Retardation, 43(5), 322–333. doi:10.1352/0047-6765(2005)43 16131229

[CIT0010] Carmeli, E., Cahana, C., & Merrick, J. (2004). The assimilation of assistive technology in residential care centers for people with intellectual disabilities. Scientific World, 4, 178–185. doi:10.1100/tsw.2004.16 PMC5956475 15105957

[CIT0011] Carter, N., Bryant-Lukosius, D., DiCenso, A., Blythe, J., & Neville, A. J. (2014). The use of triangulation in qualitative research. Oncology Nursing Forum, 41(5), 545–547. doi:10.1188/14.ONF.545-547 25158659

[CIT0012] Cavallo, F., Aquilano, M., & Arvati, M. (2015). An ambient assisted living approach in designing domiciliary services combined with innovative technologies for patients with Alzheimer’s disease: A case study. American Journal of Alzheimer’s Disease and other Dementias, 30(1), 69–77. doi:10.1177/1533317514539724 PMC10852970 24951634

[CIT0014] Centers for Medicare and Medicaid Services. (2020a). *Guidance for infection control and prevention of coronavirus disease 2019 (COVID-19) in nursing homes*. https://www.cdc.gov/coronavirus/2019-nCoV/hcp/index.html

[CIT0015] Chen, A. T., Ryskina, K. L., & Jung, H. Y. (2020). Long-term care, residential facilities, and COVID-19: An overview of federal and state policy responses. Journal of the American Medical Directors Association, 21(9), 1186–1190. doi:10.1016/j.jamda.2020.07.001 32859298 PMC7334928

[CIT0016] Chen, Y., & Schulz, P. J. (2016). The effect of information communication technology on reducing social isolation in the elderly: A systematic review. Journal of Medical Internet Research, 18(1), e18. doi:10.2196/jmir.4596 26822073 PMC4751336

[CIT0017] Conroy, K., Krishnan, S., Mittelstaedt, S., & Patel, S. (2020). Technological advancements to address elderly loneliness: Practical considerations and community resilience implications for COVID-19 pandemic. Working with Older People, 24(4), 257–264. doi:10.1108/wwop-07-2020-0036.Technological 33679208 PMC7932172

[CIT0018] Cornwell, E. Y., & Waite, L. (2009). Social disconnectedness, perceived isolation, and health among older adults. Journal Health Social Behavior, 50(1), 31–48. doi:10.1177/002214650905000103 PMC2756979 19413133

[CIT0019] Courtenay, K., & Perera, B. (2020). COVID-19 and people with intellectual disability: Impacts of a pandemic. Irish Journal of Psychological Medicine, 37(3), 231–236. doi:10.1017/ipm.2020.45 32404232 PMC7287305

[CIT0020] Demiris, G., Parker Oliver, D. R., Hensel, B., Dickey, G., Rantz, M., & Skubic, M. (2008). Use of videophones for distant caregiving: An enriching experience for families and residents in long-term care. Journal of Gerontological Nursing, 34(7), 50–55. doi:10.3928/00989134-20080701-02 18649824

[CIT0021] Dinapoli, E. A., Wu, B., & Scogin, F. (2014). Social isolation and cognitive function in Appalachian older adults. Research on Aging, 36(2), 161–179. doi:10.1177/0164027512470704 25650688

[CIT0022] Edelman, L. S., McConnell, E. S., Kennerly, S. M., Alderden, J., Horn, S. D., & Yap, T. L. (2020). Mitigating the effects of a pandemic: Facilitating improved nursing home care delivery through technology. JMIR Aging, 3(1), 1–7. doi:10.2196/20110 PMC7252197 32412909

[CIT0023] Elias, S. M. S . (2018). Prevalence of loneliness, anxiety, and depression among older people living in long-term care: A review. International Journal of Care Scholars, 1(1), 39–43. https://journals.iium.edu.my/ijcs/index.php/ijcs/article/view/44

[CIT0024] Elza, R., Barton, C., & Fehskens, C. (2017). Reducing social isolation in affordable senior housing using voice assistant technology. AARP Foundation. https://www.aarp.org/aarp-foundation/

[CIT0025] Gallistl, V., Seifert, A., & Kolland, F. (2021). COVID-19 as a “Digital Push?” research experiences from long-term care and recommendations for the post-pandemic era. Frontiers in Public Health, 9(May), 1–4. doi:10.3389/fpubh.2021.660064 PMC8141793 34041216

[CIT0026] Ho, D. K . (2018). Voice-controlled virtual assistants for older people with visual impairment. Eye, 32, 53–54. doi:10.1038/eye.2017.165 28776586 PMC5770711

[CIT0027] Holt-Lunstad, J., Smith, T. B., Baker, M., Harris, T., & Stephenson, D. (2015). Loneliness and social isolation as risk factors for mortality: A meta-analytic review. Perspectives on Psychological Science, 10(2), 227–237. doi:10.1177/1745691614568352 25910392

[CIT0028] Ibarra, F., Baez, M., Cernuzzi, L., & Casati, F. (2020). A systematic review on technology supported interventions to improve old-age social wellbeing: Loneliness, social isolation, and connectedness. Journal of Healthcare Engineering, 2020, 14. doi:10.1155/2020/2036842 PMC7374226 32765823

[CIT0029] Jones, V. K . (2019). Experiencing voice-activated artificial intelligence assistants in the home: A phenomenological approach. University of Nebraska. https://digitalcommons.unl.edu/cehsdiss/348/

[CIT0030] Kakulla, B. N . (2020). Older adults keep pace on tech usage. AARP. https://www.aarp.org/research/topics/technology/info-2019/2020-technology-trends-older-americans.html

[CIT0031] Kim, S . (2021). Exploring how older adults use a smart speaker-based voice assistant in their first interactions: Qualitative study. JMIR mHealth and uHealth, 9(1), e20427. doi:10.2196/20427 33439130 PMC7840274

[CIT0032] Kirkham, C., & Lesser, B. (2020). Special report: Pandemic exposes systemic staffing problems at U.S. nursing homes. Reuters. https://www.reuters.com/article/us-health-coronavirus-nursinghomes-speci/special-report-pandemic-exposes-systemic-staffing-problems-at-u-s-nursing-homes-idUSKBN23H1L9

[CIT0033] Kluck, J. P., Stoyanova, F., & Krämer, N. C. (2021). Putting the social back into physical distancing: The role of digital connections in a pandemic crisis. International Journal of Psychology, 56(4), 594–606. doi:10.1002/ijop.12746 33615476 PMC8013576

[CIT0034] Kolanowski, A., Boltz, M., Galik, E., Gitlin, L. N., Kales, H. C., Resnick, B., Haitsma, K. S., Van Knehans, A., Sutterlin, J. E., Sefcik, J. S., Liu, W., Petrovsky, V., Massimo, L., Gilmore-bykovskyi, A., Macandrew, M., Brewster, G., Nalls, V., Jao, Y., Duffort, N., & Health, O. (2019). Determinants of behavioral and psychological symptoms of dementia: A scoping review of the evidence. Nursing Outlook, 65(5), 515–529. doi:10.1016/j.outlook.2017.06.006.Determinants PMC6579119 28826872

[CIT0035] Lau-Ng, R., Caruso, L. B., & Perls, T. T. (2020). COVID-19 deaths in long-term care facilities: A critical piece of the pandemic puzzle. Journal of the American Geriatrics Society, 68(9), 1895–1898. doi:10.1111/jgs.16669 32501537 PMC7300761

[CIT0036] Liddell, K., Ruck, A., Holland, A., & Huppert, J. (2021). Isolating residents including wandering residents in care and group homes: Medical ethics and English law in the context of Covid-19. International Journal of Law and Psychiatry, 74, 101649. doi:10.1016/j.ijlp.2020.101649 33418151 PMC7705359

[CIT0037] Lincoln, Y. S. & Guba, E. G. (1985). Naturalistic inquiry. Sage Publications.

[CIT0038] MacLeod, S., Tkatch, R., Kraemer, S., Fellows, A., McGinn, M., Schaeffer, J., & Yeh, C. (2021). COVID-19 era social isolation among older adults. Geriatrics, 6(2), 52. doi:10.3390/geriatrics6020052 34069953 PMC8162327

[CIT0039] Marshall, C., & Rossman, G. (1999). Designing qualitative research (3rd ed.). Sage.

[CIT0040] Masina, F., Orso, V., Pluchino, P., Dainese, G., Volpato, S., Nelini, C., Mapelli, D., Spagnolli, A., & Gamberini, L. (2020). Investigating the accessibility of voice assistants with impaired users: Mixed methods study. Journal of Medical Internet Research, 22(9), e18431. doi:10.2196/18431 32975525 PMC7547392

[CIT0041] McArthur, C., Saari, M., Heckman, G., Wellens, N., Weir, J., Hebert, P., Turcotte, L., Jbilou, J., & Hirdes, J. P. (2021). Evaluating the effect of covid-19 pandemic lockdown on longterm care residents’ mental health: A data-driven approach in New Brunswick. Journal of the American Medical Directors Association, 22(1), 187–192. doi:10.1016/j.jamda.2020.10.028 33232682 PMC7587131

[CIT0042] Mo, S., & Shi, J. (2020). The psychological consequences of the Covid-19 on residents and staff in nursing homes. Work, Aging and Retirement, 6(4), 254–259. doi:10.1093/workar/waaa021 34192005 PMC7665707

[CIT0043] Moyle, W., Jones, C., Murfield, J., Dwan, T., & Ownsworth, T. (2018). “We don’t even have Wi-Fi”: A descriptive study exploring current use and availability of communication technologies in residential aged care. Contemporary Nurse, 54(1), 35–43. doi:10.1080/10376178.2017.1411203 29185380

[CIT0044] Nelson, M., & Poulin, K. (1997). Method of constructivist inquiry. In T. Sexton & B. Griffin (Eds.), Constructivist thinking in counseling practice (pp. 157–173). Teachers College Press.

[CIT0045] O’Brien, K., Liggett, A., Ramirez-Zohfeld, V., Sunkara, P., & Lindquist, L. A. (2020). Voice controlled intelligent personal assistants to support aging in place. Journal of the American Geriatrics Society, 68(1), 176–179. doi:10.1111/jgs.16217 31617581

[CIT0046] O’Rourke, H. M., & Sidani, S. (2017). Definition, determinants, and outcomes of social connectedness for older adults: A scoping review. Journal of Gerontological Nursing, 43(7), 43–52. doi:10.3928/00989134-20170223-03 28399313

[CIT0047] Powell, K. R., Alexander, G. L., Madsen, R., & Powell, K. R. (2019). A national assessment of access to technology among nursing home residents: A secondary analysis corresponding author. Journal of Materials Research, 2(1), 1–9. doi:10.2196/11449 PMC6714997 31518285

[CIT0048] Pradhan, A., Mehta, K., & Findlater, L. (2018). “Accessibility came by accident”: Use of voice controlled intelligent personal assistants by people with disabilities. Proceedings of the 2018 CHI Conference on Human Factors in Computing Systems Paper No.: 459. Association for Computing Technology. P. 13. doi:10.1145/3173574.3174033

[CIT0049] Sheerman, L., Marston, H. R., Musselwhite, C., & Morgan, D. (2020). COVID-19 and the secret virtual assistants: The social weapons for a state of emergency. Emerald Open Research, 2, 19. doi:10.35241/emeraldopenres.13571.1

[CIT0050] Simard, J., & Volicer, L. (2020). Loneliness and isolation in long-term care and the COVID-19 pandemic. Journal of the American Geriatrics Society, 21(7), 19–21. doi:10.1016/j.jamda.2020.05.006 PMC7205644 32505516

[CIT0051] Smith, M. L., Steinman, L. E., & Casey, E. A. (2020). Combatting social isolation among older adults in a time of physical distancing: The COVID-19 social connectivity paradox. Frontiers in Public Health, 8, 1–9. doi:10.3389/fpubh.2020.00403 32850605 PMC7396644

[CIT0052] Tanis, E. S., Palmer, S. B., Wehmeyer, M. L., Stock, S., & Bishop, B. (2014). A self-report computer-based survey of technology use by people with intellectual and developmental disabilities. Intellectual and Developmental Disabilities, 50(1), 53–68. doi:10.1352/1934-9556-50.1.53.A PMC3990586 22316226

[CIT0053] Thompson, D. C., Barbu, M. G., Beiu, C., Popa, L. G., Mihai, M. M., Berteanu, M., & Popescu, M. N. (2020). The impact of covid-19 pandemic on long-term care facilities worldwide: An overview on international issues. Biomed Research International. 2020. doi:10.1155/2020/8870249 PMC7656236 33204723

[CIT0054] Valtorta, N. K., Kanaan, M., Gilbody, S., Ronzi, S., & Hanratty, B. (2016). Loneliness and social isolation as risk factors for coronary heart disease and stroke: Systematic review and meta-analysis of longitudinal observational studies. Heart, 102(13), 1009–1016. doi:10.1136/heartjnl-2015-308790 27091846 PMC4941172

[CIT0055] White, E. M., Fox, T., & Reddy, A. (2021). Front-line nursing home staff experiences during the COVID-19 pandemic. Journal of the American Medical Directors Association, 22(1), 199–203. doi:10.1016/j.jamda.2020.11.022 33321076 PMC7685055

[CIT0056] Wu, B . (2020). Social isolation and loneliness among older adults in the context of COVID-19: A global challenge. Global Health Research and Policy, 5(1), 154–156. doi:10.1186/s41256-020-00154-3 PMC7272234 32514427

[CIT0057] Zavaleta, D., Samuel, K., & Mills, C. T. (2017). Measures of social isolation. Social Indicators Research, 13(1), 367–291. doi:10.1007/s11205-016-1252-2

